# A Rare Case of Primary Mycobacterium tuberculosis Myositis of the Thigh in an Immunocompetent Infant

**DOI:** 10.7759/cureus.35983

**Published:** 2023-03-10

**Authors:** Hadi A Helali, Sarah Gharaibeh, Zainab A Malik

**Affiliations:** 1 Department of Pediatrics, Al Jalila Children’s Speciality Hospital, Dubai, ARE; 2 Department of Pediatrics, Mediclinic City Hospital, Dubai, ARE; 3 Department of Pediatric Infectious Diseases, Mediclinic City Hospital, Dubai, ARE; 4 College of Medicine, Mohammed Bin Rashid University of Medicine and Health Sciences, Dubai, ARE

**Keywords:** extrapulmonary tuberculosis (eptb), anti-tuberculosis therapy, mtb (mycobacterium tuberculosis), infant, myositis, mycobacterium tuberculosis

## Abstract

*Mycobacterium tuberculosis* (MTB) infection is a serious health condition that affects individuals of all age groups. Although MTB infections are more common in immunocompromised patients, they are frequently diagnosed in healthy individuals without apparent risk factors. Extrapulmonary infection is an uncommon manifestation of MTB infection, especially infection of the musculoskeletal system. Here, we present a rare case of a five-month-old immunocompetent infant who presented with a progressively enlarging swelling of the thigh without any other symptoms. After further evaluation, a diagnosis of primary MTB myositis of the thigh was made, which was treated successfully with first-line anti-tuberculosis therapy for nine months. This case report highlights the need to consider MTB infection in infants and children with unusual clinical findings. Due to its nonspecific symptoms and difficulty in diagnosis, clinicians need to maintain a high index of clinical suspicion for MTB infection, even in infants without risk factors for exposure.

## Introduction

*Mycobacterium tuberculosis* (MTB) infection is considered one of the leading causes of morbidity and mortality in the world [1], with over 240,000 deaths annually among children due to infections or complications related to MTB infection [2]. It is estimated that around 10.4 million persons worldwide are infected with this pathogen every year according to the World Health Organization (WHO) annual report in 2016 [2]. The WHO also reported that half a million MTB infections occur in individuals younger than 15 years of age in 2012 [3], comprising 10% of MTB infections globally [2]. While MTB infections among adults occur primarily in the respiratory system, extrapulmonary infections are more frequent in children [4]. In adults, it is estimated that only 3% of patients with extrapulmonary MTB have musculoskeletal infections [5], and, out of those, only around 3% have primary MTB myositis [6]. Here, we present the case of a five-month-old previously healthy infant who presented with MTB myositis of the thigh which was successfully treated with first-line anti-tuberculosis (TB) therapy.

## Case presentation

A previously healthy five-month-old girl was referred to the Pediatric Infectious Disease Clinic in view of a two-month history of progressively worsening swelling over her right thigh. There was no reported tenderness or restriction in leg movement. A review of systems was negative for fever, cough, weight loss, or night sweats.

The infant was born at term in the United Arab Emirates (UAE) following an uncomplicated pregnancy and delivery and had received her primary vaccinations as per the national immunization schedule. Infants born in the UAE routinely receive the hepatitis B vaccine in the thigh and the Bacille Calmette-Guerin (BCG) vaccine in the left deltoid prior to discharge from the hospital. She was previously healthy, growing very well, up to date for routine vaccines, and was meeting all age-appropriate developmental milestones at presentation. The family denied any travel history or exposure to any relatives or house workers with fever, weight loss, or cough which suggests exposure to active tuberculosis, and the child’s siblings had received all their early childhood vaccinations.

On examination, the infant was well-appearing and afebrile, had the BCG scar, and had a firm swelling noted anterolaterally on her right thigh. There was no overlying erythema, ulceration, tenderness, or fluctuance, and a full range of motion at the right hip and knee. There was no inguinal or systemic lymphadenopathy, and the remaining physical examination was unremarkable.

**Figure 1:**
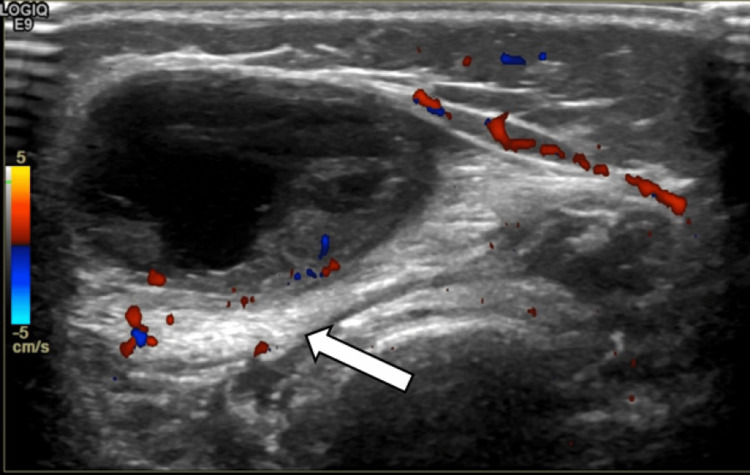
Ultrasound of the right thigh showing an intramuscular heterogenous ovoid cystic mass with septation and internal soft tissue, measuring 41 mm × 13 mm × 24 mm. Surrounding tissue exhibits increased vascularity, however, the mass itself does not appear to have internal vascularity. There is evidence of posterior acoustic enhancement (arrow).

Thigh ultrasound scan (USS) (Figure 1) showed a subcutaneous oval complex mass measuring approximately 41 mm × 13 mm × 24 mm. It was a predominantly cystic lesion, with low-grade echoes and a hypervascular wall.

**Figure 2:**
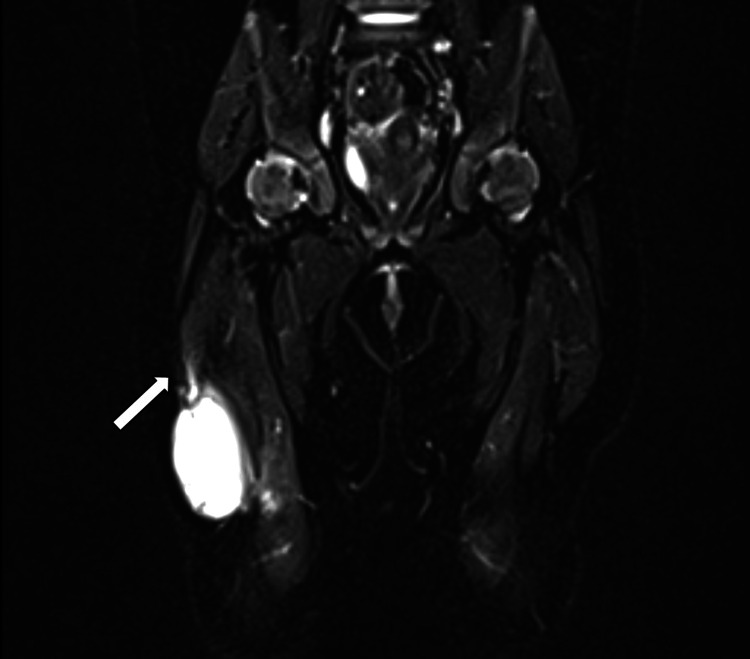
T2-weighted MRI of the thigh, coronal view. A cystic mass is visualized along the anterolateral aspect of the distal thigh, measuring 40 mm × 14 mm × 22 mm in craniocaudal, transverse, and anteroposterior dimensions. The mass has a broad base against the vastus lateralis with infiltration into the muscle (arrow).

**Figure 3:**
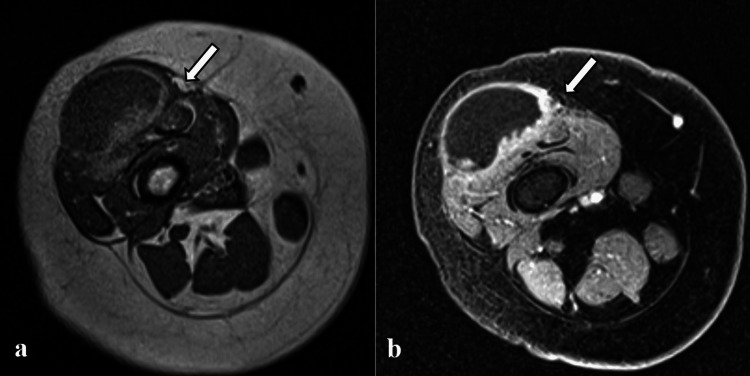
T1 MRI of the thigh, axial view, before (a) and after (b) contrast. Before contrast administration (a), the cystic mass is visualized over the anterolateral thigh, with a solid component evident along its posterior aspect (arrow). After contrast administration (b), there is an enhancement of the capsular walls and the solid component (arrow).

Initial laboratory parameters, including inflammatory markers (C-reactive protein (CRP), procalcitonin, and erythrocyte sedimentation rate (ESR)), were within normal limits. Magnetic resonance imaging (MRI) with contrast under general anesthesia (Figure 2) showed a large cystic mass along the anterolateral aspect of the distal right thigh, infiltrating the vastus lateralis muscle and the distal end of the rectus femoris muscle. It measured 40 mm × 14 mm × 22 mm. The mass was mainly cystic with a solid component along its posterior aspect (Figure 3a). After contrast administration, there was an enhancement of the entire capsular wall and the solid component, along with restricted diffusion noted in the solid component and only the adjacent component of the capsular wall (Figure 3b). Neither neurovascular bundle involvement nor evidence of lymphadenopathy was visualized. However, a few prominent lymph nodes were seen in both inguinal areas. The adjacent joint spaces were unremarkable, without signs of effusion.

A USS-guided biopsy of the cystic lesion was obtained. Histopathology was negative for malignant cells but showed necrotizing granulomas. Smear microscopy for acid-fast bacilli was negative. However, the tuberculosis polymerase chain reaction (TB-PCR) on the biopsy fluid was positive for the MTB complex. Because Quantiferon TB assay is unreliable in children <2 years, a purified protein derivative was placed, which demonstrated high reactivity with a 25 mm induration after 72 hours of placement. Chest X-ray and abdominal ultrasound showed no evidence of disseminated disease.

Given her young age and high risk of disseminated infection, the infant was empirically started on four-drug anti-TB therapy (rifampin, isoniazid, pyrazinamide, and ethambutol) while awaiting TB culture. She tolerated the medications very well and showed a significant reduction in thigh mass within two weeks of starting treatment. After 56 days of incubation, TB culture was reported as having no growth. However, due to the high likelihood of tuberculous myositis in this young infant, the risk of dissemination, and her excellent response to the anti-TB regimen, medications were continued as planned after discussions with the family.

After completing two months of the four-drug therapy, treatment was de-escalated to isoniazid and rifampicin. Repeat USS six months after initiating treatment showed a significant reduction in the size of the mass with reduced wall thickening and clearance of internal debris.

After nine months of treatment, the lesion had completely normalized on examination leaving no signs of asymmetry between the legs. A repeat MRI with contrast obtained nine months after the initial scan showed a resolution of active disease with the disappearance of the cyst. Her anti-TB therapy was discontinued, and she remains under close follow-up, with no signs of recurrence 18 months after discontinuing therapy.

## Discussion

Despite advances in modern medicine, infection with MTB remains a serious cause of morbidity and mortality among all age groups globally [1]. Establishing a diagnosis of MTB infection in infants and children is fraught with challenges including nonspecific signs and symptoms of infection, difficulty in collecting samples for microbiological confirmation, paucibacillary disease in children, and challenges with growing MTB on culture media [1,4,7,8]. All these factors require a high index of suspicion for MTB infection in children presenting with unusual clinical findings, such as isolated swelling or weight loss only [9]. A systemic review showed that most of the cases of primary MTB myositis found were in adults and only 15% of these patients were children [9].

Primary MTB myositis is a rare presentation of infection in children and is reported to have good clinical outcomes [9,10]. The rarity of MTB myositis in general is attributed to the nature of the organism and the physiology of the skeletal muscle. TB bacilli thrive and proliferate within the reticuloendothelial cells and need oxygen to grow [10]. Skeletal muscle has few reticuloendothelial cells and usually contains a low reservoir of oxygen. With strenuous use, skeletal muscle cells can accumulate high concentrations of lactic acid, which makes it an unfavorable ground for the growth of MTB [11]. Some authors have proposed that primary MTB myositis might occur after direct inoculation of the organism following an injection into the area [12]. The rationale is that this would allow an entry site for MTB into the unhospitable area of the muscle and trauma related to the injection would introduce inflammatory cells into the area, making it favorable for the growth of the organism [13]. This theory is supported by the fact that MTB myositis in adults is more common in males, especially those with a history of preceding trauma [10]. The case was suspected to have primary MTB myositis of the thigh because one of the authors dealt with a similar case of isolated TB myositis after vaccination but in an older child.

Primary MTB myositis is frequently reported in the literature among adults and less common among children. Bae et al. [13] reported MTB myositis in the arm of an immunocompetent 17-month-old toddler following infection with the hepatitis A vaccine. Similarly, Malik et al. [7] reported MTB myositis in a nine-month-old infant following routine infant vaccinations. Both reports suggest direct inoculation during routine immunization as the underlying cause of infection. Due to the rarity of this diagnosis and unusual clinical presentation, such children benefit from a multidisciplinary team approach. With the widespread availability of TB-PCR, timely and accurate diagnosis can be obtained, and treatment initiated while awaiting confirmation by MTB culture after six to eight weeks [3,7].

## Conclusions

Infection of skeletal muscles with MTB is a rare but serious cause of morbidity and disability in children. Available reports suggest excellent outcomes in children with MTB myositis treated with a prolonged course of first-line anti-MTB therapy. TB-PCR is an important modality for timely diagnosis and treatment, with a high sensitivity and specificity for the detection of the MTB complex. Due to its nonspecific symptoms, unusual presentation, rarity in occurrence, and difficulty in diagnosis, clinicians need to maintain a high index of clinical suspicion for MTB myositis when infants and children present with unusual clinical findings.
